# Simplified Diagnosis of Critical Illness Polyneuropathy in Patients with Prolonged Mechanical Ventilation: A Prospective Observational Cohort Study

**DOI:** 10.3390/jcm9124029

**Published:** 2020-12-13

**Authors:** Chul Jung, Nak-Jun Choi, Won Jun Kim, Yoon Mok Chun, Hak-Jae Lee, Tae Hyun Kim, Sae Rom Pak, Jung Hoon Lee, Suk-Kyung Hong, Won Kim

**Affiliations:** 1 Department of Rehabilitation Medicine, Asan Medical Center, University of Ulsan College of Medicine, 88 Olympic-ro 43-gil, Songpa-gu, Seoul 05505, Korea; speciron90@gmail.com (C.J.); happyslow87@naver.com (W.J.K.); hooni8107@hanmail.net (J.H.L.); 2 Division of Trauma Surgery, Department of Surgery, Korea University Guro Hospital, Seoul 08308, Korea; njchoi@korea.ac.kr; 3 Wooridul Spine Hospital, Seoul 07505, Korea; ch-mogy@hanmail.net; 4 Division of Acute Care Surgery, Department of Surgery, Asan Medical Center, University of Ulsan College of Medicine, Seoul 05505, Korea; lhj206@hanmail.net (H.-J.L.); saerom_pak@amc.seoul.kr (S.R.P.); 5 Department of Surgery, Seoul Medical Center, Seoul 02053, Korea; present8518@naver.com

**Keywords:** critical illness polyneuropathy, simplified diagnosis, electrophysiology, clinical outcomes, mechanical ventilation

## Abstract

Background: Although early identification of critical illness polyneuropathy (CIP) is necessary, the established diagnostic criteria have several limitations in the intensive care unit (ICU) setting. The purpose of this study was to define simplified diagnostic criteria of CIP that best predict clinical outcomes. Methods: This prospective, single-center study included 41 ICU patients with prolonged mechanical ventilation (≥21 days). We applied three different sets of diagnostic criteria (combining the results of the Medical Research Council (MRC) sum score and nerve conduction studies (NCS)) for CIP in order to identify the criteria with the best predictive power for clinical outcomes. Results: The simplified diagnosis of CIP meeting the criteria, i.e., that the MRC sum score < 48 and amplitudes of the tibial and sural nerve < 80% of the lower limit of normal, showed the strongest association with 0 ventilator-free days at day 60 (odds ratio, 6.222; *p* = 0.029). Conclusions: The diagnostic criteria combining the MRC sum score and the tibial and the sural NCS were identified as the simplified criteria of CIP that best predicted the clinical outcomes. The implementation of these simplified criteria may allow for early identification of CIP in the ICU, thereby contributing to prompt interventions for patients with a poor prognosis.

## 1. Introduction

As intensive care unit (ICU) mortality is decreasing, there is a growing concern about the survivorship of patients with a critical illness [1]. Survivors of critical illness may experience marked disability and impairment in cognition, mental health, and physical function. ICU-acquired weakness (ICUAW) is the most common presentation of physical impairment and it occurs in more than 25% of ICU survivors [2,3,4,5,6]. One of the factors strongly associated with development of ICUAW is prolonged mechanical ventilation [6,7,8]. Patients requiring prolonged mechanical ventilation were reported to develop ICUAW at a higher incidence (43% of patients after 7 days of mechanical ventilation) [5]. ICUAW commonly manifests in one of three ways: critical illness polyneuropathy (CIP), critical illness myopathy (CIM), or severe disused muscle atrophy [2,7,9,10].

CIP is a sensory-motor axonal polyneuropathy affecting not only limb muscles but also respiratory muscles [9,11,12,13]. Patients with CIP have been reported to have worse clinical outcomes than patients with CIM or disuse atrophy [9,14]. Therefore, early identification and therapeutic intervention for CIP are necessary for maximizing the recovery of critically ill patients. The diagnosis of CIP is established based mainly on electrophysiological tests. The diagnostic criteria using full electrophysiological tests were proposed and evaluated in previous studies [15,16]. Full electrophysiological tests include nerve conduction studies (NCS) in conjunction with needle electromyography and repetitive nerve stimulation bilaterally performed on the upper and lower limbs. However, there are several difficulties involved in performing the full electrophysiological tests in the ICU, which include the time-consuming nature of the tests, electrical interference caused by medical devices, and the patients’ noncooperation [2,7,16,17,18].

Therefore, previous studies suggested using a single NCS as a simplified diagnostic tool for CIP prior to performing the full electrophysiological tests [11,16,17,19]. The best diagnostic accuracy for patients at risk for CIP was achieved with peroneal motor NCS [11,17] alone or combined with sural sensory NCS [16,19]. The sensitivity and diagnostic accuracy of peroneal motor NCS was superior to that of the other single NCS measurements; however, the possibility that peroneal motor NCS can be affected by peroneal nerve dysfunction, irrespective of CIP, was presented as a limitation of the previous studies [11,16,17,19]. This condition includes compressive peroneal neuropathy, tissue edema, and extensor digitorum brevis muscle atrophy related to advanced age [11,16]. In addition, previous studies never addressed comparisons of the strength of the association between the simplified tests of CIP and the clinical outcomes.

The aim of this study was to define simplified diagnostic criteria of CIP that best predicted clinical outcomes in patients with prolonged mechanical ventilation. We examined three different diagnostic criteria for CIP. The simplified criteria of CIP with the best predictive power were pursued through a comparison of the strength of the association between criteria and clinical outcomes. In addition, we investigated the clinical characteristics, risk factors, and respiratory involvement of CIP diagnosed using the simplified criteria.

## 2. Materials and Methods

### 2.1. Study Design

We performed a prospective observational cohort study between October 2016 and April 2018 in two surgical ICUs at a tertiary hospital in Seoul, Korea with more than 2000 beds. The two surgical ICUs had 14 and 12 beds, respectively, and both of them were staffed by surgical ICU physicians with a nurse to patient ratio of 1:2.

This preplanned study is a sub-analysis of a trial that investigated the clinical characteristics and risk factors of critically ill surgical patients dependent on prolonged mechanical ventilation. The trial was registered at the Clinical Research Information Service of South Korea (KCT0005387). The trial protocol and consent forms were reviewed and approved by the ethics committee of our institution (IRB No. 2016-0799). Patients eligible for the trial, those who required mechanical ventilation for at least three days, were screened. Written informed consent was obtained from all patients or their authorized representatives prior to trial enrollment.

### 2.2. Study Population

This study included patients who were ≥18 years old and were dependent on mechanical ventilation for ≥21 days. Patients with pre-existing peripheral and central nervous system disorders that significantly reduced their motor function or patients with emergent major surgery after the NCS were excluded from the study. Other exclusion criteria were patient/representative refusal to participate and terminal conditions.

### 2.3. Study Procedures

The patients were assessed by the Medical Research Council (MRC) sum score and were evaluated by NCS performed on the unilateral limbs and phrenic nerve. We applied three different diagnostic criteria for CIP to these patients and investigated the simplified diagnostic criteria with the best predictive power for clinical outcomes.

#### 2.3.1. MRC Sum Score

The MRC sum score was used for the clinical quantification of muscle strength. To assess the score, the primary physician tested bilateral six muscle groups—shoulder abduction, elbow flexion, wrist extension, hip flexion, knee extension, and foot dorsiflexion—on the same day as the NCS [2,15,20]. The MRC sum score was counted as <48 if the volitional technique was limited by the patients’ unconsciousness and/or noncooperation. The patients’ alertness or sedation level was determined with the Richmond Agitation Sedation Scale. The physician was blinded to the NCS data.

#### 2.3.2. NCS

Fifty patients who required mechanical ventilation for at least 21 days received the NCS. The NCS recordings were performed by skilled neurophysiologists using a Medelec Synergy N2 (Carefusion, San Diego, CA, USA). In consideration of the symmetric pattern of CIP, the recordings were conducted on the unilateral upper and lower limbs [11,18,20,21]. The tested side for each patient was chosen according to its better motor function and physical access to the nerves. The reason for performing the test on the side with better strength was to rule out focal neuropathy that existed independently of CIP.

The tests included conduction studies of three motor nerves (ulnar, peroneal and tibial) and two sensory nerves (ulnar and sural). The tested nerves were selected based on previous studies [11,16,17,19,21]. Compound muscle action potentials (CMAPs) were recorded from the ulnar, peroneal and tibial nerves. They were elicited by stimulation at distal and proximal sites in order to exclude the presence of a conduction block and to calculate the conduction velocity. For the ulnar nerve, CMAP was recorded from surface electrodes placed on the abductor digiti minimi muscle with stimulation at the wrist and elbow. For the peroneal nerve, it was recorded from electrodes placed on the extensor digitorum brevis muscle with stimulation at the ankle and below the fibular head. For the tibial nerve, it was recorded from electrodes placed on the abductor hallucis muscle with stimulation at the medial malleolus and popliteal fossa. Sensory nerve action potentials (SNAPs) were recorded from the ulnar and sural nerves. For the ulnar nerve, SNAP was recorded from surface electrodes placed on the fifth finger with stimulation at the wrist. For the sural nerve, it was recorded from electrodes placed on the lateral malleolus with stimulation at the calf. For all nerves, supramaximal stimulation was given to obtain the best CMAP or SNAP amplitudes. Reduced CMAP or SNAP amplitudes were defined when they were less than 80% of the lower limit of the normal range for our laboratory [15,16].

In addition to NCS performed on the limbs, phrenic motor NCS was done bilaterally. CMAP was recorded from the surface electrode placed on the diaphragm at the xyphoid process with stimulation at the posterior and inferior border of the sternocleidomastoid muscle. The reference electrode was placed over the anterior costal margin 16 cm from the recording electrode [12,13,18,22]. After supramaximal stimulation, the best CMAP amplitude was obtained for each side and the higher amplitude was selected. If phrenic motor NCS could not be performed due to technical problems, or the obtained amplitudes were obscured, such patients or amplitudes were excluded from the analysis. For the phrenic nerve, a reduced CMAP amplitude was defined when it was less than 0.2 mV [22].

All recordings were reviewed for quality control and interpreted by two authors (CJ and WK) with expert knowledge in electrophysiologic tests. The authors were blinded to the MRC sum score at the time of their review and interpretation.

### 2.4. Diagnostic Criteria for CIP

We applied three different diagnostic criteria of CIP to the patients (Table 1). They were diagnosed with CIP if they met Criteria A, B, or C. We established Criteria A based on accepted standards and previous studies [11,15,16]. Criteria B and C were established as simplified compared to Criteria A. Criteria A and B included the MRC sum score as well as the NCS data; however, Criteria C only included the NCS results. To be specific, Criteria A included reduced CMAP and SNAP amplitudes in ≥2 motor and sensory nerves, which are located in the unilateral upper and lower limbs, respectively, while meeting the ICUAW diagnostic criteria with an MRC sum score <48 [5,23]. Criteria B included the MRC sum score cutoff for ICUAW and reduced amplitudes in the tibial and sural nerves, which are located only in the unilateral lower limbs. Criteria C were defined only as reduced amplitudes in the tibial and sural nerves. The tibial and sural nerves were selected because they are less likely to develop focal neuropathy.

### 2.5. Clinical Data Collection and Outcomes

The following clinical data were collected: age, sex, comorbidities, past medical history, admission type, and nutritional and surgical status at the time of ICU admission. Nutritional status was assessed with the Academy of Nutrition and Dietetics-American Society for Parenteral and Enteral Nutrition (AND-A.S.P.E.N.) consensus [24]. We also collected data including the performed procedures and medication administered during the ICU admission period and the Acute Physiology and Chronic Health Evaluation II (APACHE II) score for critical illness severity. Duration of mechanical ventilation, ventilator-free days at day 60 (VFDs-60), ICU length of stay, ICU-free days at day 60 (IFDs-60), hospital length of stay, and ICU mortality were collected as outcome data. We defined VFDs-60 as the number of days out of 60 that the patient was alive and weaned from mechanical ventilation. IFDs-60 was defined as the number of days out of 60 that the living patient was out of the ICU. The 60-day time horizon was chosen to calculate VFDs based on published literature and this study’s medical context (i.e., when most patients were expected to be extubated or deceased) [25,26,27,28].

The primary outcome was the simplified diagnostic criteria of CIP which is best predictive for clinical outcomes, evaluated through a comparison of the strength of the association between the three simplified criteria of CIP (Criteria A, B, or C) and the clinical outcome (VFDs-60). The clinical characteristics and risk factors of the patients with a simplified diagnosis of CIP were investigated as secondary outcomes. We also explored the association of reduced phrenic CMAP amplitude with a simplified CIP diagnosis and with the VFDs-60.

### 2.6. Sample Size Calculation and Statistical Analysis

This study is a preplanned sub-analysis of a trial conducted during a one-year period. After the data of the patients admitted to the two surgical ICUs were reviewed, four to five patients a month were estimated to be eligible for this study. Therefore, the final cohort size was targeted at 50 patients.

All statistical analyses were performed using PASW Statistics 18 (SPSS Inc., Chicago, IL, USA). We defined a *p* value < 0.05 as a statistically significant cut-off value. Continuous variables are presented as mean ± standard deviation or median (interquartile range (IQR)), and categorical variables are presented as counts (percentage). We used Pearson’s χ^2^ test, and Fisher’s exact test to evaluate the strength of the association between Criteria A, B, or C and the VFDs-60. In the analysis of the association between the VFDs-60 and the other clinical variables, the VFDs-60 were categorized into two groups: 0 and ≥1. The Mann-Whitney U test and Fisher’s exact test were used to compare variables between the patients with and without a simplified diagnosis of CIP. Multivariate logistic regression analysis with stepwise fashioned variable selection was used to identify the factors associated with an increased risk of the simplified CIP diagnosis. Variables with a *p* value < 0.2 in the univariate analysis were considered as possible risk factors. All possible risk factors were included in the multivariate analysis. The association of a reduced phrenic CMAP amplitude with a simplified CIP diagnosis and with VFDs-60 was investigated using Fisher’s exact test.

## 3. Results

**Figure 1:**
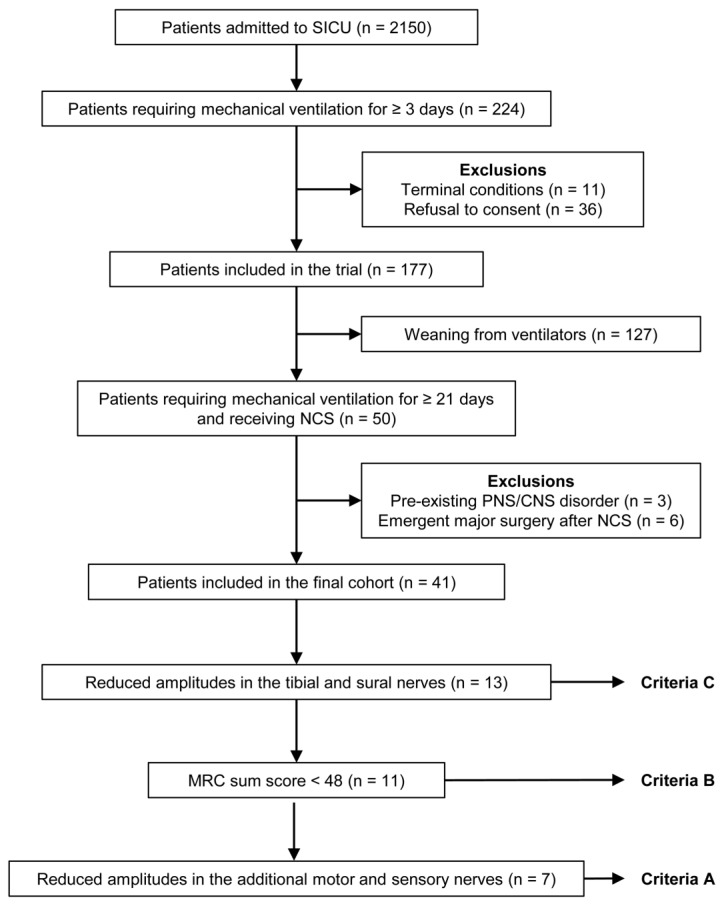
Flow diagram for patient inclusion and exclusion.

A total of 2150 critically ill surgical patients were admitted to the two surgical ICUs during the study period. Among them, 224 patients required mechanical ventilation for at least three days and were eligible for the trial. Eleven patients with terminal conditions and 36 patients who declined to participate were excluded. A total of 177 patients were included in the trial. In the trial population, 50 patients with prolonged mechanical ventilation (≥21 days) were included in this study and received NCS at the time of inclusion. Patients with pre-existing peripheral and central nervous system disorders significantly reducing their motor function (*n* = 3) and those with emergent major surgery after the NCS (*n* = 6) were excluded. As a result, the final cohort included 41 patients (Figure 1). Five patients were not responsive to verbal commands (Richmond Agitation Sedation Scale ≤ −3) at the time of the NCS and therefore scored <48 on the MRC sum score.

### 3.1. Patient Characteristics

The clinical characteristics and outcomes of the total cohort are presented in Table 2. According to the admission type, seven (17.1%) patients were admitted to the ICU for postoperative monitoring, while the other patients were admitted requiring intensive treatment primarily for septic shock (53.7% of the patients). Their median APACHE II score was 27 (20–32.5). Seventeen (41.5%) patients were identified as malnourished at the time of ICU admission. The median duration of mechanical ventilation, length of stay in the ICU and in the hospital, were 46 (31–64.5), 48 (34–64), and 67 (52–106) days, respectively. A total of 12 patients died in the ICU.

### 3.2. Simplified Diagnosis of CIP

#### 3.2.1. Comparison of the Strength of the Association between the Simplified Criteria and the VFDs-60

Seven (17.1%), 11 (26.8%), and 13 (31.7%) patients met Criteria A, B, and C, respectively, and were diagnosed with CIP (Figure 1). Table 3 shows the association of a simplified CIP diagnosis according to each Criteria with a VFDs-60 of zero. In the simplified CIP diagnosis using Criteria B, the strongest association with a VFDs-60 of zero was identified with the lowest *p* value and highest odds ratio (odds ratio [OR], 6.222; *p* = 0.029). As the criteria become progressively more simplified (from Criteria A to Criteria C), sensitivity for a VFDs-60 of zero increased from 29.4% to 47.1%, and specificity for a VFDs-60 of zero decreased from 91.7% to 79.2%.

#### 3.2.2. Clinical Characteristics and Risk Factors of Patients with CIP According to Criteria B

The comparison of clinical characteristics and outcomes between the patients with a simplified diagnosis of CIP using Criteria B and those without the diagnosis is presented in Table 4. For all clinical outcomes, the patients with CIP exhibited poorer outcomes than those without CIP. Specifically, the CIP group patients had lower VFDs-60 and IFDs-60, and higher ICU mortality. Except for ICU mortality (*p* = 0.247), differences between the two groups were statistically significant. The patients with CIP also had low tibial and phrenic CMAP and sural SNAP amplitudes on NCS.

Using univariate logistic regression analysis, the association of clinical variables with the simplified diagnosis of CIP according to Criteria B was assessed (Table 5). In multivariate analysis using forward stepwise variable selection, the risk of CIP was higher in patients with severe malnutrition (OR, 240.554; 95% CI, 4.333–13,355.084) and a longer duration of steroid use (OR, 1.119; 95% CI, 1.027–1.219) after adjusting for other factors. When backward stepwise selection was applied, the same factors remained significant. Duration of dialysis was associated with a nonsignificant increase in the risk of CIP (OR, 1.149; 95% CI, 0.997–1.324).

### 3.3. Phrenic Motor NCS

The phrenic motor NCS could be performed bilaterally for only 20 patients. The test was performed unilaterally in 20 other patients and could not be performed on the remaining patient. Unilateral tests were conducted due to a surgical site or presence of central venous catheters blocking access to one side, or the patient’s refusal. In addition, even the obtained phrenic CMAP amplitudes were obscured in five patients. As a result, 35 patients were included in the analysis of phrenic motor NCS. The reduced phrenic CMAP showed a significant association with an increased risk of CIP according to Criteria B (OR, 17.250; *p* value = 0.003) (Table 6). Despite no statistical significance, a reduced phrenic CMAP amplitude was associated with a VFDs-60 of zero (OR, 3.188; *p* value = 0.151).

## 4. Discussion

The diagnosis of CIP conventionally requires NCS bilaterally performed on the upper and lower limbs, namely Criteria A in this study [11,15,16,18]. In addition, it is also necessary to perform repetitive nerve stimulation and needle electromyography for the exclusion of neuromuscular junction disorders and CIM. This full electrophysiological test is reported to take about 90 min for patients on mechanical ventilation, and therefore it has only limited use in the ICU [11,16,23]. Interference caused by electrical devices in the ICU and patients’ unconsciousness and noncooperation are also obstacles to completing this test. Although it is limited in an ICU setting, early identification of patients with CIP is still an important issue in that CIP is associated with poor outcomes of patients with a critical illness [2,7,9]. Early diagnosis of CIP may also lead to prompt interventions. To be specific, the patients with CIP usually have difficulty with weaning from mechanical ventilation, and thus, the physician can carefully attempt the weaning process in these patients. Patients with CIP may also benefit from early and active rehabilitation, which is associated with better clinical outcomes [2,9,29,30,31], and from risk factor management for preventing the progression of CIP. Therefore, we established three simplified criteria for the diagnosis of CIP and investigated which criteria had a significant association with clinical outcomes, including VFDs-60. To our knowledge, this is the first study to investigate and define the simplified diagnostic criteria of CIP with the best predictive power for clinical outcomes.

Since sensory nerves are spared in neuromuscular junction disorders and CIM, we tried to diagnose CIP solely with NCS performed on the motor and sensory nerves. If there is an abnormality in sensory NCS, CIP can be roughly diagnosed without performing repetitive nerve stimulation and needle electromyography. We also performed NCS unilaterally because CIP symmetrically involves multiple nerves [9,16,18,21]. Based on this line of thinking, we set Criteria A as reduced CMAP and SNAP amplitudes of ≥2 motor and sensory nerves in the unilateral limbs, and an MRC sum score <48 being satisfied simultaneously. We established Criteria B as more simplified than Criteria A. We selected the tibial and sural nerves for Criteria B because CIP is known to preferentially affect the lower limbs [9]. In the previous literature, peroneal motor NCS alone or combined with sural sensory NCS was identified as having the best sensitivity and diagnostic accuracy [11,16,17,19]. However, we chose the tibial nerve for several reasons. First, the previous study reported the tibial nerve had a comparable sensitivity, specificity, and diagnostic accuracy for the diagnosis of CIP to those of the peroneal nerve (sensitivity, 94% vs. 94%; specificity, 69% vs. 74%; diagnostic accuracy, 0.8315 vs. 0.8611). The feasibility of the tibial nerve, however, was higher than that of the peroneal nerve (87% vs. 84%) [16]. Secondly, peroneal nerve dysfunction such as peroneal neuropathy irrespective of CIP, was reported to be relatively common in patients who are critically ill [32,33]. Simplified criteria including the tibial nerve would have advantages for avoiding this issue. Lastly, the time-saving nature and ease of performing the peroneal motor NCS are also true for the tibial motor NCS. Although not formally measured, the time needed for performing unilateral tibial motor and sural sensory NCS, which are included in Criteria B and C, is about 10 min [11,16]. The tibial and sural NCS are also not difficult to perform. Future studies should perform a comparison test with the results from the peroneal nerve. Criteria C, the most simplified set of criteria, was defined only with the tibial motor and the sural sensory NCS. Clinical weakness assessed by the MRC sum score was excluded from Criteria C. If only electrophysiological features of CIP are considered (like Criteria C), a similar concept of a probable diagnosis of CIP is reached, as previously suggested [10].

Then, we investigated different sets of simplified diagnostic criteria of CIP, looking for the one that best predicts the clinical outcomes. This was done by comparing the strength of the association between the criteria and the clinical outcome (VFDs-60). As a result, Criteria B showed the strongest association with a VFDs-60 of zero. Criteria B, though it was more simplified than Criteria A, showed a stronger association with poor clinical outcomes. Criteria B had a significantly higher sensitivity than that of Criteria A, while both of them similar specificities. This meant that Criteria B selected appropriate nerves and tested the nerves efficiently. We also identified that Criteria B was superior to Criteria C. This implied that the MRC sum score representing clinical weakness needs to be included in the diagnostic criteria. Supporting this finding, recent studies revealed that a low MRC sum score and reduced CMAP were independently associated with 1-year and 5-year mortality [4,34,35]. Despite having no statistical significance (*p* = 0.075), Criteria C was also associated with an increased risk of 0 VFDs-60. This is consistent with previous studies reporting that an electrophysiological abnormality by itself is significantly associated with a poor clinical outcome [34,36]. In consideration of the fact that the MRC sum score requires patients’ cooperation, it is advisable to apply Criteria B to critically ill patients who can cooperate, whereas Criteria C may have its value in critically ill patients in the acute stage, who are generally sedated and unconscious.

To define the simplified diagnostic criteria of CIP with the best predictive power for clinical outcomes, the strength of the association between the criteria and a VFDs-60 of zero was evaluated by Pearson’s χ^2^ test and Fisher’s exact test. For these tests, the VFDs-60 was dichotomized into values = 0 and ≥ 1. After the simplified criteria were defined, the difference of VFDs-60 between the patients with and without CIP was evaluated by the Mann-Whitney U test. Likewise, we used two different statistical methods for the VFDs-60, based on the fact that VFD is a continuous variable; however, it also has the nature of a binary variable [28].

We also explored the clinical characteristics and outcomes of the patients diagnosed with CIP using Criteria B. Eleven of 41 patients developed CIP, and this prevalence (26.8%) is consistent with previous reports [11,16,19]. The clinical outcomes were significantly different between the patients with and without CIP. To be specific, the VFDs-60 and IFDs-60 were significantly smaller in the patients with CIP. In particular, the VFDs-60 showed a statistically significant difference of about 12 days, which can be considered as a clinically significant difference based on previous studies using VFDs-60 [25,26]. These findings are supported by several studies reporting that CIP is associated with a longer duration of mechanical ventilation and ICU stay [16,37,38]. Meanwhile, ICU mortality showed no statistical significance, which was in contrast with previous studies [16,37,39]. Considering the mortality in patients with CIP (45.5%) was almost double that in those without CIP (23.3%), we suspect a lack of statistical power related to the small study population and number of events may be affecting this finding.

In addition, two significant factors associated with an increased risk of CIP were identified in the final model derived from the multivariate logistic regression analysis. The first factor, severe malnutrition at the time of ICU admission, was significantly associated with an increased risk of CIP in patients with three weeks of mechanical ventilation. The odds ratio (240.554) would have been exaggerated, since only one patient with severe malnutrition did not develop CIP. This is an intuitive finding when we consider that patients with severe malnutrition are likely to be more critically ill. In addition, more active use of total parenteral nutrition might be associated with the development of polyneuropathy in these severely malnourished patients. Total parenteral nutrition was reported to increase the risk of CIP by worsening disturbances in the microcirculation [37]. The other factor, steroid use, has also been reported by several previous studies to increase the risk of ICUAW [2,9,40,41,42]. The duration of steroid use was probably identified as a factor associated with an increased risk of CIP in this study since it reflects the severity of the critical illness and provokes hyperglycemia.

In previous studies, CIP was reported to weaken respiratory strength by affecting the phrenic nerve [2,9,12,13]. This study identified that a simplified diagnosis of CIP was associated with a reduced phrenic CMAP amplitude. This association could provide an explanation for why a simplified CIP diagnosis was related to prolonged mechanical ventilation and ICU stay. A reduced phrenic CMAP amplitude also showed a tendency to increase the risk of 0 VFDs-60 by three-fold; however, this was not statistically significant. This could be partly explained by several studies reporting that there was no significant correlation between respiratory function and the results of phrenic motor NCS in healthy subjects [22,43,44]. These studies suggested that weakness of the diaphragm might be compensated for by the activity of other respiratory muscles. In addition, the difficulties of performing bilateral phrenic motor NCS would have contributed to the weak association between the phrenic CMAP amplitude and 0 VFDs-60. We could perform bilateral phrenic motor NCS on only 20 of 41 patients due to a surgical site, presence of central venous catheters, or the patient’s refusal. Moreover, the phrenic CMAP amplitudes obtained in five patients were obscured, probably due to the normally small phrenic CMAP amplitudes and interference from electrical devices in the ICU. All of these technical problems limit the clinical value of phrenic motor NCS in the ICU setting.

There are several limitations to this study. First, we could not completely exclude all of the patients with pre-existing peripheral polyneuropathy because we did not perform baseline NCS when the patients were admitted to the ICU. However, in the actual clinical setting, it is difficult to identify pre-existing peripheral polyneuropathy in ICU patients. Second, since repetitive nerve stimulation and needle electromyography were not performed, we were not able to screen for the coexistence of neuromuscular junction disorders or CIM. We could not also investigate the sensitivity and diagnostic accuracy of the simplified diagnostic criteria for CIP diagnosed with full electrophysiological tests. Third, we included only a small and highly selected (old age and high severity of critical illness) sample of subjects because this study was performed in two ICUs at one hospital. The small number of patients could have limited the interpretation of the results by multivariate analysis. The age of the patient and the severity of a critical illness in this study population were notably high, which might be attributed to the characteristics of ICUs at our institution and the patients’ dependence on prolonged mechanical ventilation. This limits the generalizability of our study’s results to other ICUs. Further multi-centered studies of a larger sample size would be helpful to validate the results of this study. Fourth, the primary outcome was assessed after dichotomization of the VFDs-60. The dichotomization could have statistically exaggerated the clinically irrelevant difference. This issue might be partially supplemented by the finding that patients with CIP showed not only statistically, but also clinically, significant smaller VFDs-60 than the patients without CIP.

## 5. Conclusions

Diagnostic criteria including the MRC sum score, tibial motor NCS, and sural sensory NCS were identified as the simplified criteria for CIP that best predicted the clinical outcomes. The prevalence of CIP was 26.8% and the risk factors were identified as severe malnutrition at ICU admission and a longer duration of steroid use. CIP was also significantly associated with reduced phrenic CMAP amplitudes. The implementation of simplified criteria may allow for early identification of CIP in the ICU, thereby contributing to prompt intervention for patients with a poor prognosis.

## Figures and Tables

**Table 1:** Simplified diagnostic criteria for critical illness polyneuropathy.

Criteria A	Criteria B	Criteria C
MRC sum score <48	MRC sum score <48	
CMAP amplitudes <80% of the lower limit of normal in ≥2 nerves	CMAP amplitudes <80% of the lower limit of normal in the tibial nerve	CMAP amplitudes <80% of the lower limit of normal in the tibial nerve
SNAP amplitudes <80% of the lower limit of normal in ≥2 nerves	SNAP amplitudes <80% of the lower limit of normal in the sural nerve	SNAP amplitudes <80% of the lower limit of normal in the sural nerve

MRC, Medical Research Council; CMAP, compound muscle action potential; SNAP, sensory nerve action potential.

**Table 2:** Clinical characteristics and outcomes of the patients.

	*n* = 41
**Clinical Characteristics**	
Sex, male sex, *n* (%)	32 (78.0)
Age, years, median (IQR)	70 (61.5–78)
Reason for ICU admission, *n* (%)	
––Septic shock	22 (53.7)
––Hypovolemic shock	5 (12.2)
––Cardiogenic shock	1 (2.4)
––Respiratory failure	4 (9.8)
––Altered mental status	2 (4.9)
––Postoperative monitoring	7 (17.1)
Postoperative admission, *n* (%)	32 (78.0)
APACHE II score, median (IQR)	27 (20–32.5)
Nutritional status at ICU admission, *n* (%)	
––Not malnourished	24 (58.5)
––Moderate malnutrition	12 (29.3)
––Severe malnutrition	5 (12.2)
Diabetes mellitus, *n* (%)	13 (31.7)
Chronic kidney disease, *n* (%)	7 (17.1)
History of chemotherapy, *n* (%)	6 (14.6)
History of transplantation, *n* (%)	6 (14.6)
**Clinical outcomes**	
Duration of mechanical ventilation, days, median (IQR)	46 (31–64.5)
ICU length of stay, days, median (IQR)	48 (34–64)
Hospital length of stay ^a^, days, median (IQR)	67 (52–106)
ICU mortality, *n* (%)	12 (29.3)

^a^ Hospital length of stay was counted after ICU admission. IQR, interquartile range; ICU, intensive care unit; APACHE II, Acute Physiology and Chronic Health Evaluation II.

**Table 3:** Association between the simplified criteria for CIP and VFDs-60.

		VFDs-60 ^a^ = 0	VFDs-60 ^a^ ≥ 1	*p* Value (OR)	Sensitivity	Specificity
Criteria A	CIP +	5 (71.4)	2 (28.6)			
	CIP −	12 (35.3)	22 (64.7)	0.105 (4.583)	29.4%	91.7%
Criteria B	CIP +	8 (72.7)	3 (27.3)			
	CIP −	9 (30.0)	21 (70.0)	0.029 (6.222)	47.1%	87.5%
Criteria C	CIP +	8 (61.5)	5 (38.5)			
	CIP −	9 (32.1)	19 (67.9)	0.075 (3.378)	47.1%	79.2%

^a^ VFDs-60 were defined as the number of days out of 60 that the patient was alive and off the ventilator. CIP, critical illness polyneuropathy; VFDs-60, ventilator-free days at day 60; OR, odds ratio.

**Table 4:** Clinical characteristics and outcomes of patients with CIP according to Criteria B.

Criteria B	CIP +, *n* = 11	CIP −, *n* = 30	*p* Value
**Clinical characteristics**			
Age, years, mean ± SD	66.36 ± 7.90	68.97 ± 12.66	0.237
Reason for ICU admission, *n* (%)			
––Septic shock	8 (72.7)	14 (46.7)	
––Other than septic shock	3 (27.3)	16 (53.3)	0.138
APACHE II score, mean ± SD	27.18 ± 6.75	26.10 ± 9.21	0.761
Nutritional status at ICU admission, *n* (%)			
––Not malnourished	3 (27.2)	21 (70.0)	
––Moderate malnutrition	4 (36.4)	8 (26.7)	
––Severe malnutrition	4 (36.4)	1 (3.3)	0.011
Diabetes mellitus, *n* (%)	4 (36.4)	9 (30.0)	0.719
Chronic kidney disease, *n* (%)	4 (36.4)	3 (10.0)	0.069
History of chemotherapy, *n* (%)	2 (18.2)	4 (13.3)	0.651
History of transplantation, *n* (%)	4 (36.4)	2 (6.7)	0.035
Duration ^a^ of dialysis, days, mean ± SD	13.82 ± 11.65	6.67 ± 8.25	0.091
Duration ^a^ of NM blocker use, days, mean ± SD	0.64 ± 1.43	0.73 ± 2.20	0.988
Duration ^a^ of steroid use, days, mean ± SD	12.55 ± 10.66	4.00 ± 6.57	0.007
**Clinical outcomes**			
VFDs-60 ^b^, days, mean ± SD	3.18 ± 8.95	15.67 ± 13.52	0.012
IFDs-60 ^b^, days, mean ± SD	3.09 ± 9.92	14.03 ± 12.68	0.015
ICU mortality, *n* (%)	5 (45.5)	7 (23.3)	0.247
**NCS results**			
Tibial CMAP amplitudes, mV, mean ± SD	1.94 ± 1.25	7.03 ± 3.96	<0.001
Sural SNAP amplitudes, μV, mean ± SD	0.94 ± 2.09	5.68 ± 5.61	0.009
Phrenic CMAP amplitudes ^c^, mV, mean ± SD	0.11 ± 0.14 (***n*** = 8)	0.50 ± 0.33 (***n*** = 27)	0.001

^a^ Duration was counted as the number of days in the period from ICU admission to the NCS. ^b^ VFDs-60 and IFDs-60 were defined as the number of days out of 60 that the patient was alive and off the ventilator and out of the ICU, respectively. ^c^ Data for 35 patients were analyzed. CIP, critical illness polyneuropathy; SD, standard deviation; ICU, intensive care unit; APACHE II, Acute Physiology and Chronic Health Evaluation II; NM, neuromuscular; VFDs-60, ventilator-free days at day 60; IFDs-60, intensive care unit-free days at day 60; NCS, nerve conduction studies; CMAP, compound motor action potential; SNAP, sensory nerve action potential.

**Table 5:** Logistic regression analysis of patients with CIP according to Criteria B.

Variables	Univariate Analysis		Multivariate Analysis	
	OR (95% CI)	*p* Value	OR (95% CI)	*p* Value
Age	0.981 (0.925–1.040)	0.521		
Diabetes mellitus	1.333 (0.311–5.716)	0.698		
Chronic kidney disease	5.143 (0.928–28.500)	0.061		
History of chemotherapy	1.444 (0.225–9.269)	0.698		
History of transplantation	8.000 (1.210–52.884)	0.031		
APACHE II	1.015 (0.936–1.102)	0.717		
Reason for ICU admission				
(reference: other than septic shock)				
––Septic shock	3.048 (0.674–13.773)	0.148		
Nutritional status				
(reference: Not malnourished)				
––Moderate malnutrition	3.500 (0.637–19.238)	0.150	1.716 (0.197–14.950)	0.625
––Severe malnutrition	28.000 (2.291–342.150)	0.009	240.554 (4.333–13355.084)	0.007
Duration ^a^ of dialysis, days	1.081 (1.003–1.166)	0.043	1.149 (0.997–1.324)	0.055
Duration ^a^ of NM blocker use, days	0.974 (0.675–1.406)	0.890		
Duration ^a^ of steroid use, days	1.119 (1.027–1.219)	0.010	1.119 (1.027–1.219)	0.010

^a^ Duration was counted as the number of days in the period from ICU admission to the nerve conduction study. CIP, critical illness polyneuropathy; OR, odds ratio; CI, confidence interval; APACHE II, Acute Physiology and Chronic Health Evaluation II; ICU, intensive care unit; NM, neuromuscular.

**Table 6:** Association of phrenic CMAP amplitude (*n* = 35) with CIP according to Criteria B and VFDs-60.

		Phrenic CMAP Amplitude < 0.2 mV	Phrenic CMAP Amplitude ≥ 0.2 mV	*p* Value (OR)
Criteria B	CIP +	6 (60.0)	2 (8.0)	
	CIP −	4 (40.0)	23 (92.0)	0.003 (17.250)
	VFDs-60 ^a^ = 0	6 (60.0)	8 (32.0)	
	VFDs-60 ^a^ ≥ 1	4 (40.0)	17 (68.0)	0.151 (3.188)

^a^ VFDs-60 was defined as the number of days out of 60 that the patient was alive and off the ventilator. CMAP, compound motor action potential; CIP, critical illness polyneuropathy; VFDs-60, ventilator-free days at day 60; OR, odds ratio.

## References

[1] Iwashyna T.J., Cooke C.R., Wunsch H., Kahn J.M. (2012). Population burden of long-term survivorship after severe sepsis in older Americans. J. Am. Geriatr. Soc..

[2] Vanhorebeek I., Latronico N., Van den Berghe G. (2020). ICU-acquired weakness. Intensive Care Med..

[3] Fan E., Dowdy D.W., Colantuoni E., Mendez-Tellez P.A., Sevransky J.E., Shanholtz C., Himmelfarb C.R., Desai S.V., Ciesla N., Herridge M.S. (2014). Physical complications in acute lung injury survivors: A two-year longitudinal prospective study. Crit. Care Med..

[4] Hermans G., Van Mechelen H., Clerckx B., Vanhullebusch T., Mesotten D., Wilmer A., Casaer M.P., Meersseman P., Debaveye Y., Van Cromphaut S. (2014). Acute outcomes and 1-year mortality of intensive care unit-acquired weakness. A cohort study and propensity-matched analysis. Am. J. Respir. Crit. Care Med..

[5] Fan E., Cheek F., Chlan L., Gosselink R., Hart N., Herridge M.S., Hopkins R.O., Hough C.L., Kress J.P., Latronico N. (2014). An official American Thoracic Society Clinical Practice guideline: The diagnosis of intensive care unit-acquired weakness in adults. Am. J. Respir. Crit. Care Med..

[6] Saccheri C., Morawiec E., Delemazure J., Mayaux J., Dubé B.P., Similowski T., Demoule A., Dres M. (2020). ICU-acquired weakness, diaphragm dysfunction and long-term outcomes of critically ill patients. Ann. Intensive Care.

[7] Jolley S.E., Bunnell A.E., Hough C.L. (2016). ICU-acquired weakness. Chest.

[8] De Jonghe B., Bastuji-Garin S., Durand M.C., Malissin I., Rodrigues P., Cerf C., Outin H., Sharshar T., Groupe de Réflexion et d’Etude des Neuromyopathies En Réanimation (2007). Respiratory weakness is associated with limb weakness and delayed weaning in critical illness. Crit. Care Med..

[9] Kress J.P., Hall J.B. (2014). ICU-acquired weakness and recovery from critical illness. N. Engl. J. Med..

[10] Latronico N., Bolton C.F. (2011). Critical illness polyneuropathy and myopathy: A major cause of muscle weakness and paralysis. Lancet Neurol..

[11] Latronico N., Bertolini G., Guarneri B., Botteri M., Peli E., Andreoletti S., Bera P., Luciani D., Nardella A., Vittorielli E. (2007). Simplified electrophysiological evaluation of peripheral nerves in critically ill patients: The Italian multi-centre CRIMYNE study. Crit. Care.

[12] Zifko U.A., Zipko H.T., Bolton C.F. (1998). Clinical and electrophysiological findings in critical illness polyneuropathy. J. Neurol. Sci..

[13] Santos P.D., Teixeira C., Savi A., Maccari J.G., Neres F.S., Machado A.S., de Oliveira R.P., Ribeiro M., Rotta F.T. (2012). The critical illness polyneuropathy in septic patients with prolonged weaning from mechanical ventilation: Is the diaphragm also affected? A pilot study. Respir. Care.

[14] Guarneri B., Bertolini G., Latronico N. (2008). Long-term outcome in patients with critical illness myopathy or neuropathy: The Italian multicentre CRIMYNE study. J. Neurol. Neurosurg. Psychiatry.

[15] Stevens R.D., Marshall S.A., Cornblath D.R., Hoke A., Needham D.M., de Jonghe B., Ali N.A., Sharshar T. (2009). A framework for diagnosing and classifying intensive care unit-acquired weakness. Crit. Care Med..

[16] Moss M., Yang M., Macht M., Sottile P., Gray L., McNulty M., Quan D. (2014). Screening for critical illness polyneuromyopathy with single nerve conduction studies. Intensive Care Med..

[17] Latronico N., Nattino G., Guarneri B., Fagoni N., Amantini A., Bertolini G., GiVITI Study Investigators (2014). Validation of the peroneal nerve test to diagnose critical illness polyneuropathy and myopathy in the intensive care unit: The multicentre Italian CRIMYNE-2 diagnostic accuracy study. F1000Research.

[18] Preston D.C., Shapiro B.E. (2020). Electromyography and Neuromuscular Disorders: Clinical-Electrophysiologic-Ultrasound Correlations.

[19] Kelmenson D.A., Quan D., Moss M. (2018). What is the diagnostic accuracy of single nerve conduction studies and muscle ultrasound to identify critical illness polyneuromyopathy: A prospective cohort study. Crit. Care.

[20] Vanpee G., Hermans G., Segers J., Gosselink R. (2014). Assessment of limb muscle strength in critically ill patients: A systematic review. Crit. Care Med..

[21] Wieske L., Verhamme C., Witteveen E., Bouwes A., Dettling-Ihnenfeldt D.S., van der Schaaf M., Schultz M.J., van Schaik I.N., Horn J. (2015). Feasibility and diagnostic accuracy of early electrophysiological recordings for ICU-acquired weakness: An observational cohort study. Neurocrit. Care.

[22] Vincent M., Court-Fortune I., Costes F., Antoine J.C., Camdessanché J.P. (2019). Phrenic nerve conduction in healthy subjects. Muscle Nerve.

[23] Leijten F.S., Poortvliet D.C., de Weerd A.W. (1997). The neurological examination in the assessment of polyneuropathy in mechanically ventilated patients. Eur. J. Neurol..

[24] White J.V., Guenter P., Jensen G., Malone A., Schofield M., Academy Malnutrition Work Group, ASPEN Malnutrition Task Force, ASPEN Board of Directors (2012). Consensus statement: Academy of Nutrition and Dietetics and American Society for Parenteral and Enteral Nutrition: Characteristics recommended for the identification and documentation of adult malnutrition (undernutrition). JPEN J. Parenter. Enteral Nutr..

[25] Bein T., Weber-Carstens S., Goldmann A., Müller T., Staudinger T., Brederlau J., Muellenbach R., Dembinski R., Graf B.M., Wewalka M. (2013). Lower tidal volume strategy (≈3 ml/kg) combined with extracorporeal CO_2_ removal versus ‘conventional’ protective ventilation (6 ml/kg) in severe ARDS: The prospective randomized Xtravent-study. Intensive Care Med..

[26] Mekontso Dessap A., Katsahian S., Roche-Campo F., Varet H., Kouatchet A., Tomicic V., Beduneau G., Sonneville R., Jaber S., Darmon M. (2014). Ventilator-associated pneumonia during weaning from mechanical ventilation: Role of fluid management. Chest.

[27] Contentin L., Ehrmann S., Giraudeau B. (2014). Heterogeneity in the definition of mechanical ventilation duration and ventilator-free days. Am. J. Respir. Crit. Care Med..

[28] Bodet-Contentin L., Frasca D., Tavernier E., Feuillet F., Foucher Y., Giraudeau B. (2018). Ventilator-Free Day Outcomes Can Be Misleading. Crit. Care Med..

[29] Hermans G., De Jonghe B., Bruyninckx F., Van den Berghe G. (2014). Interventions for preventing critical illness polyneuropathy and critical illness myopathy. Cochrane Database Syst. Rev..

[30] Tipping C.J., Harrold M., Holland A., Romero L., Nisbet T., Hodgson C.L. (2017). The effects of active mobilisation and rehabilitation in ICU on mortality and function: A systematic review. Intensive Care Med..

[31] Doiron K.A., Hoffmann T.C., Beller E.M. (2018). Early intervention (mobilization or active exercise) for critically ill adults in the intensive care unit. Cochrane Database Syst. Rev..

[32] Wiis J., Qvist J. (1999). Peroneal nerve paresis after long-term bed rest in intensive care patients. Article in Danish. Ugeskr. Laeger..

[33] Yoon J.S., Gwak M.S., Yang M., Kim G.S., Kwon C.H., Joh J.W., Lee S.K., Kim S.J. (2008). Peroneal neuropathy after liver transplantation. Transplant. Proc..

[34] Hermans G., Van Mechelen H., Bruyninckx F., Vanhullebusch T., Clerckx B., Meersseman P., Debaveye Y., Casaer M.P., Wilmer A., Wouters P.J. (2015). Predictive value for weakness and 1-year mortality of screening electrophysiology tests in the ICU. Intensive Care Med..

[35] Van Aerde N., Meersseman P., Debaveye Y., Wilmer A., Gunst J., Casaer M.P., Bruyninckx F., Wouters P.J., Gosselink R., Van den Berghe G. (2020). Five-year impact of ICU-acquired neuromuscular complications: A prospective, observational study. Intensive Care Med..

[36] Kelmenson D.A., Quan D., Nordon-Craft A., Malone D., Schenkman M., Moss M. (2016). Electrophysiological abnormalities can differentiate pre-hospital discharge functional status in critically ill patients with normal strength. Intensive Care Med..

[37] Garnacho-Montero J., Madrazo-Osuna J., García-Garmendia J.L., Ortiz-Leyba C., Jiménez-Jiménez F.J., Barrero-Almodóvar A., Garnacho-Montero M.C., Moyano-Del-Estad M.R. (2001). Critical illness polyneuropathy: Risk factors and clinical consequences. A cohort study in septic patients. Intensive Care Med..

[38] Garnacho-Montero J., Amaya-Villar R., García-Garmendía J.L., Madrazo-Osuna J., Ortiz-Leyba C. (2005). Effect of critical illness polyneuropathy on the withdrawal from mechanical ventilation and the length of stay in septic patients. Crit. Care Med..

[39] Leijten F.S., Harinck-de Weerd J.E., Poortvliet D.C., de Weerd A.W. (1995). The role of polyneuropathy in motor convalescence after prolonged mechanical ventilation. JAMA.

[40] Farhan H., Moreno-Duarte I., Latronico N., Zafonte R., Eikermann M. (2016). Acquired muscle weakness in the surgical intensive care unit: Nosology, epidemiology, diagnosis, and prevention. Anesthesiology.

[41] Yang T., Li Z., Jiang L., Xi X. (2018). Corticosteroid use and intensive care unit-acquired weakness: A systematic review and meta-analysis. Crit. Care.

[42] Solverson K.J., Grant C., Doig C.J. (2016). Assessment and predictors of physical functioning post-hospital discharge in survivors of critical illness. Ann. Intensive Care.

[43] Mier A., Brophy C., Moxham J., Green M. (1987). Phrenic nerve stimulation in normal subjects and in patients with diaphragmatic weakness. Thorax.

[44] Chen R., Collins S., Remtulla H., Parkes A., Bolton C.F. (1995). Phrenic nerve conduction study in normal subjects. Muscle Nerve.

